# Esophageal Microbiome in Eosinophilic Esophagitis

**DOI:** 10.1371/journal.pone.0128346

**Published:** 2015-05-28

**Authors:** J. Kirk Harris, Rui Fang, Brandie D. Wagner, Ha Na Choe, Caleb J. Kelly, Shauna Schroeder, Wendy Moore, Mark J. Stevens, Alyson Yeckes, Katie Amsden, Amir F. Kagalwalla, Angelika Zalewski, Ikuo Hirano, Nirmala Gonsalves, Lauren N. Henry, Joanne C. Masterson, Charles E. Robertson, Donald Y. Leung, Norman R. Pace, Steven J. Ackerman, Glenn T. Furuta, Sophie A. Fillon

**Affiliations:** 1 Division of Pulmonology, University of Colorado School of Medicine, Aurora, CO, United States of America; 2 Department of Biostatistics and Informatics, Colorado School of Public Health, University of Colorado, Aurora, CO, United States of America; 3 Digestive Health Institute, Section of Pediatric Gastroenterology, Hepatology and Nutrition, Children’s Hospital Colorado, Gastrointestinal Eosinophilic Diseases Program, Mucosal Inflammation Program, University of Colorado School of Medicine, Aurora, CO, United States of America; 4 Department of Pediatrics, Division of Gastroenterology, Hepatology and Nutrition, Ann & Robert H. Lurie Children’s Hospital of Chicago and Northwestern University, Chicago, IL, United States of America; 5 Department of Pediatrics, John H. Stroger Hospital of Cook County, Chicago, IL, United States of America; 6 Department of Medicine, Division of Gastroenterology & Hepatology, Northwestern University, Feinberg School of Medicine, Chicago, IL, United States of America; 7 Molecular Cellular and Developmental Biology, University of Colorado Boulder, Boulder, CO, United States of America; 8 Department of Pediatrics, National Jewish Health, Denver, CO, United States of America; 9 Department of Biochemistry and Molecular Genetics, University of Illinois at Chicago, Chicago, IL, United States of America; Cincinnati Children's Hospital Medical Center, University of Cincinnati College of Medicine, UNITED STATES

## Abstract

**Objective:**

The microbiome has been implicated in the pathogenesis of a number of allergic and inflammatory diseases. The mucosa affected by eosinophilic esophagitis (EoE) is composed of a stratified squamous epithelia and contains intraepithelial eosinophils. To date, no studies have identified the esophageal microbiome in patients with EoE or the impact of treatment on these organisms. The aim of this study was to identify the esophageal microbiome in EoE and determine whether treatments change this profile. We hypothesized that clinically relevant alterations in bacterial populations are present in different forms of esophagitis.

**Design:**

In this prospective study, secretions from the esophageal mucosa were collected from children and adults with EoE, Gastroesophageal Reflux Disease (GERD) and normal mucosa using the Esophageal String Test (EST). Bacterial load was determined using quantitative PCR. Bacterial communities, determined by 16S rRNA gene amplification and 454 pyrosequencing, were compared between health and disease.

**Results:**

Samples from a total of 70 children and adult subjects were examined. Bacterial load was increased in both EoE and GERD relative to normal subjects. In subjects with EoE, load was increased regardless of treatment status or degree of mucosal eosinophilia compared with normal. *Haemophilus* was significantly increased in untreated EoE subjects as compared with normal subjects. *Streptococcus* was decreased in GERD subjects on proton pump inhibition as compared with normal subjects.

**Conclusions:**

Diseases associated with mucosal eosinophilia are characterized by a different microbiome from that found in the normal mucosa. Microbiota may contribute to esophageal inflammation in EoE and GERD.

## Introduction

The increasing use of culture independent techniques has provided a wealth of information documenting changes in the microbiome during health and disease. The microbiome associated with mucosal surfaces has clearly emerged as an important participant in initiating or perpetuating inflammatory states. In addition, a number of factors, including medications and nutrition, may contribute to the load and composition of the intestinal microbiome.

Over the last decade, increasing clinical recognition of eosinophilic esophagitis (EoE) sparked a number of studies examining it pathogenesis. While these studies have identified a number of genes, pathogenetic pathways and co-morbid features, several factors provide a framework for considering a primary or secondary role of an altered microbiome in EoE. For example, basal zone hyperplasia in response to IL-13 may contribute to decreased mucosal barrier function as a primary factor [1], whereas esophageal eosinophils may alter the host microenvironment and perpetuate the disease and thus contribute as a secondary factor. Eosinophils are potent reservoirs of a number of anti-microbial products including granule cationic proteins, defensins and DNA-containing extracellular traps (EETs) [2–4]; release of these products may change the microbiome. Eosinophilic inflammation can disrupt esophageal motility, thus leading to stasis and proliferation of damaging species. Finally, patients with EoE restrict their diets due to food allergies, thus potentially changing the substrate for the esophageal bacteria.

On the basis of these findings, we sought to identify the esophageal microbiota in children and adults with active and inactive EoE. Identification of these microbial communities may provide clues regarding novel pathogenetic mechanisms and therapeutic targets. We hypothesized that the esophageal microbiota in the mucosa of EoE subjects was different from those with GERD and normal mucosa. Our findings support a potential role for altered microbiome in EoE with the affected mucosa harboring more *Haemophilus* compared to subjects with GERD or a normal mucosa.

## Materials and Methods

### Capture of Esophageal microbiome using the Esophageal String Test (EST)

Children and adults who were undergoing an endoscopy with biopsy to determine causes of abdominal pain, vomiting, growth failure, dysphagia or histological efficacy of EoE treatment were enrolled in this study of children and adults from Lurie Children’s Hospital/Stroger Hospital (Chicago) and Children’s Hospital Colorado (Aurora) and Northwestern Hospital (Chicago). Exclusion criteria included age less than 7 years, a history of esophageal stricture or narrowing, gelatin allergy or other co-morbidities with increased risk (bleeding diatheses, connective tissue diseases) of endoscopic complications. We included children 7 years and older because of their ability to swallow a capsule. Histories were taken to record symptoms, allergic history, family history and medications. Review of endoscopic and pathology records were performed to ensure diagnostic accuracy and determine clinical features [5, 6].

Subject diagnoses were assigned according to the following criteria based on published consensus recommendations [7–9]: *(i)*. Eosinophilic esophagitis (EoE-active)—subjects with symptoms of esophageal dysfunction, esophageal eosinophilia ≥15 eosinophils per high power field; *(ii)*. EoE-remission—subjects diagnosed with EoE as defined above who have undergone at least 8 weeks of treatment (topical steroids or dietary elimination) and who, at the time of their endoscopy, were both asymptomatic and showed evidence of histologic remission in terms of decreased esophageal eosinophil counts <15 eos/hpf. The subjects were not proton pump inhibitor-responsive esophageal eosinophilia PPI-REE. EoE is a male dominated disease, more than 70% of our EoE subjects were male *(iii)*. GERD—subjects with symptoms of vomiting or heartburn that had responded to proton pump inhibitors (PPI) and/or had an abnormal pH impedance monitor of the distal esophagus. Subjects in remission were on PPI treatment at the time of the endoscopy; *(iv)*. Normal—subjects with symptoms that lead to endoscopic testing (abdominal pain, vomiting, growth failure) and who were found to have endoscopically and histopathologically normal esophagus. Subjects in this study were not on antibiotic treatment. The night before their upper endoscopic procedure, subjects swallowed the Enterotest™ and underwent endoscopy with biopsy the following day as described previously, the EST for the microbiome identification was collected in the same mid to distal section of the esophagus as the biopsy in our previously published work [5, 6].

This study was approved by the Colorado Multiple Institutional Review Board (COMIRB), Aurora, CO, and the IRBs of the University of Illinois at Chicago, Northwestern University and Ann & Robert H. Lurie Children’s Hospital of Chicago, Chicago, IL. Written informed consent and HIPPA authorization were obtained from all participants or from parents or legal guardians of participants younger than 18 years. Assent was obtained from all participants under 18 years.

### Microbiota identification

Immediately preceding endoscopy, the EST was removed as described previously [5, 6]. A 2 cm segment of the middle esophagus was collected and snap frozen in liquid nitrogen and kept at -80°C until DNA extraction was performed. DNA from all samples was extracted using Qiagen DNAeasy Extraction Kits for blood and tissue according to manufacturer’s specifications (Qiagen, Valencia, CA). DNA was amplified in triplicate with barcoded PCR primers targeting V1/V2 (27F/338R) [10] that include sequencing adaptors, and negative controls were performed for each barcode. PCR was repeated for any sample where the negative control was positive. Amplicons were pooled after normalization of DNA concentration (SequalPrep, Invitrogen) [11], and sequenced using the Roche 454 FLX platform according to manufacturer’s specifications (Roche, Branford, CT) with an average of 695 sequences (range 112–2555), with Good's coverage of >94%. Sequence data were assigned to samples of origin using bar code sequences added during PCR, and screened for basic quality defects (sequences < 200 nucleotides in length, > 1 sequence ambiguity, best read with quality ≥ 20 over a 10-nucleotide moving window) by the program BARTAB [12]. Non-bacterial sequences were removed from datasets by requiring a close match with a bacterial rRNA secondary structure model within Infernal [13]. Sequences identified as potential chimeras by ChimeraSlayer [14] were also removed from datasets. The Ribosomal Database Project Classifier software was used to make taxonomic assignments [15]. Taxonomic information was used to construct sequence groups with identical taxonomic rank, which were used for bacterial community analyses, and to identify specific types of bacteria that were differentially present between subject groups. Sequence counts were converted to relative abundance to account for differences in sequence totals between samples [16]. Sequence data has been submitted to NCBI under the accession SRP041586. Total bacterial load was estimated using quantitative PCR (qPCR) with pan-bacterial primers that target the small subunit ribosomal RNA (SSU-rRNA) as previously described [17, 18]. Bacterial load was expressed as copy number per ng of total DNA to control for the variation in total DNA obtained for individual samples.

### Statistical analyses

Descriptive statistics for Shannon diversity index [19], a common ecological parameter, are presented. For ease of interpretation, effective number of taxa (EFT) was calculated from this index [19]. Comparisons between two groups were performed individually for each taxa using the two-part test and the negative log p-values are displayed using the Manhattan plot [20]. Principal component analysis was performed on the relative abundance data from each sample. A small constant (0.01) was added to eliminate zero values prior to the application of the centered log ratio transformation recommended for compositional data [21, 22]. All analyses were performed using SAS Version 9.3 software (SAS Institute Inc.: Cary, NC, 2011). Manhattan plots and summary figures were generated using Explicet [23] (www.explicet.org).

## Results

### Subject characteristics

Seventy subjects were recruited from Chicago, IL (n = 26) and Aurora, CO (n = 44). Details of subjects’ demographics, clinical characteristics and ethnic diversity are provided in Table 1. Some normal subjects and half of the EoE subjects were on PPI, but these were not considered a treatment for these groups, in accordance with published EoE consensus recommendations [7–9]. EoE subjects were considered “treated” when histologic remission was demonstrated on standard of care treatment including topical steroids or dietary restrictions. Treatment for subjects with GERD was prescription of PPI.

**Table 1:** Subject Characterization Summary.

Subject Groups	Number of Subjects	Number of Subjects on PPI (% of total) or specific treatment for EoE group	Age Average years (range in years)	Male Gender (% of total)	Ethnicity (% white)	Collected in Aurora CO (% of total)
**Eosinophilic esophagitis–active (untreated)**	11	5 (45.5%)	19.4 (9–49)	9 (82%)	10 (91%)	5 (45.5%)
**Eosinophilic esophagitis–remission (treated)**	26	PPI 12 (46%) Diet 14 (54%) Steroid 6 (23%) Diet and steroid 6 (23%)	17.1 (7–59)	19 (73%)	24 (92%)	12 (46%)
**Gastro-esophageal reflux disease-active (untreated)**	4	0 (0%)	29.5 (11–53)	1 (25%)	4 (100%)	2 (50%)
**Gastro-esophageal reflux disease-remission (treated)**	4	4 (100%)	13.8 (10–16)	2 (50%)	4 (100%)	4 (100%)
**Normal Esophagus**	25	11 (44%)	14.2 (11–18)	7 (28%)	19 (79%)	21 (84%)

### Bacterial load but not diversity is increased in EoE

**Fig 1:**
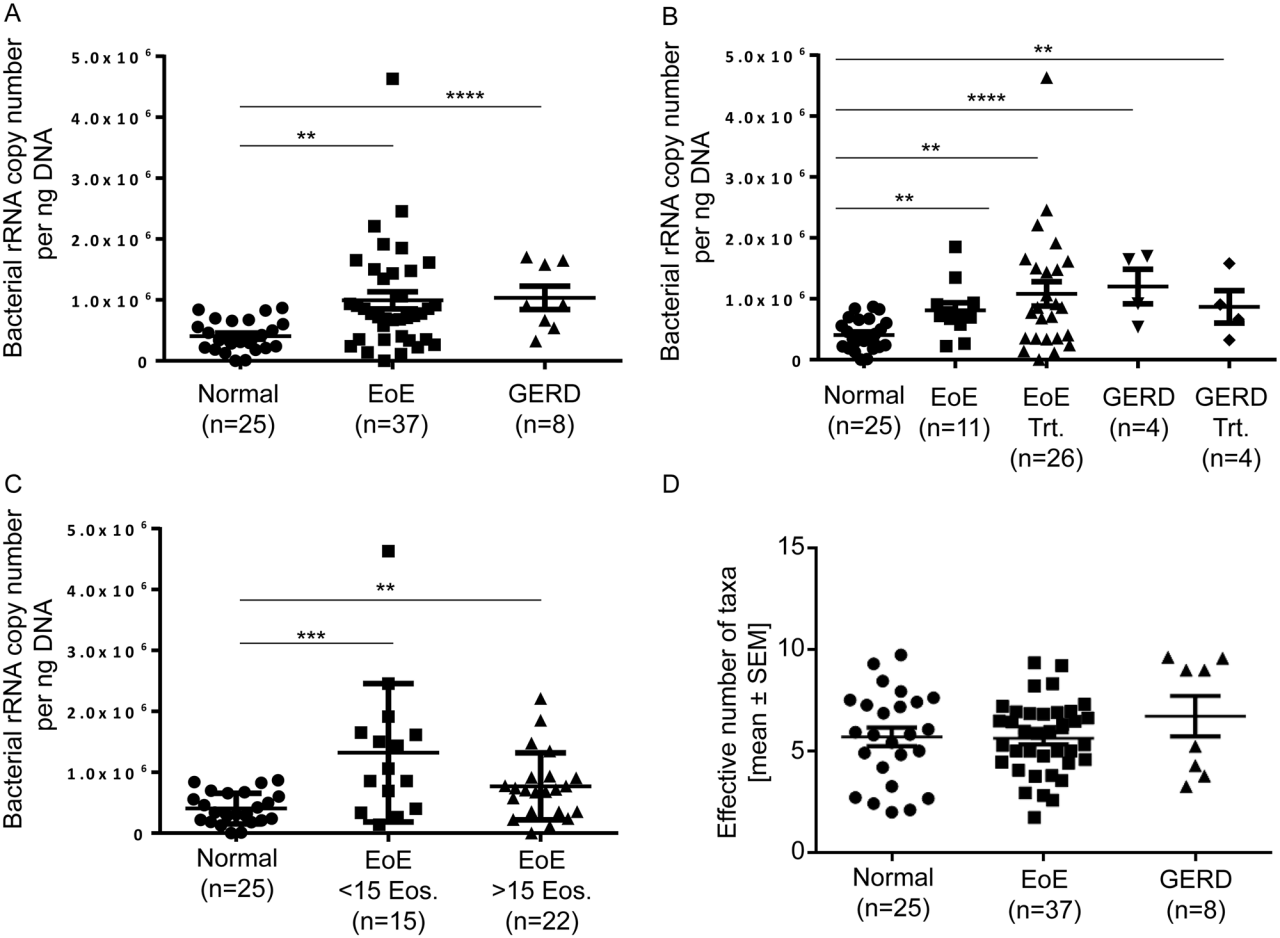
The esophageal bacterial load is increased in subjects with EoE and GERD: Analysis by 16S Q-PCR. The esophageal bacterial load was captured using the EST as previously described [5]. A. The copy number of bacteria per ng of DNA is significantly increased in subjects with EoE, and GERD as compared to Normal. B. Treatments (Trt) do not significantly change the load of bacteria in each disease. C. The disease activity or response to treatment does not significantly change the load of bacteria in EoE. D. The effective number of taxa is similar in Normal, EoE and GERD. The mean ± SEM are shown. Normal n = 25, EoE n = 37, GERD n = 8. Mean and SEM, two-sample t-test * P<0.*05*, ** P<0.*01*, *** P<0.*001*, **** P<0.*0001*.

The average bacterial load detected in all subjects with EoE was significantly greater than that determined from normal subjects (Fig 1A). These differences between disease and normal states were not influenced by treatment (Fig 1B) or disease activity (Fig 1C). By comparison, the average bacterial load found in GERD subjects was also significantly increased relative to normal esophagus control subjects (Fig 1A). No significant differences were observed for alpha diversity, represented as Effective Number of Taxa across any of the subject groups (Fig 1D).

### Effect of inflammation and treatment on esophageal phyla and genera

**Fig 2:**
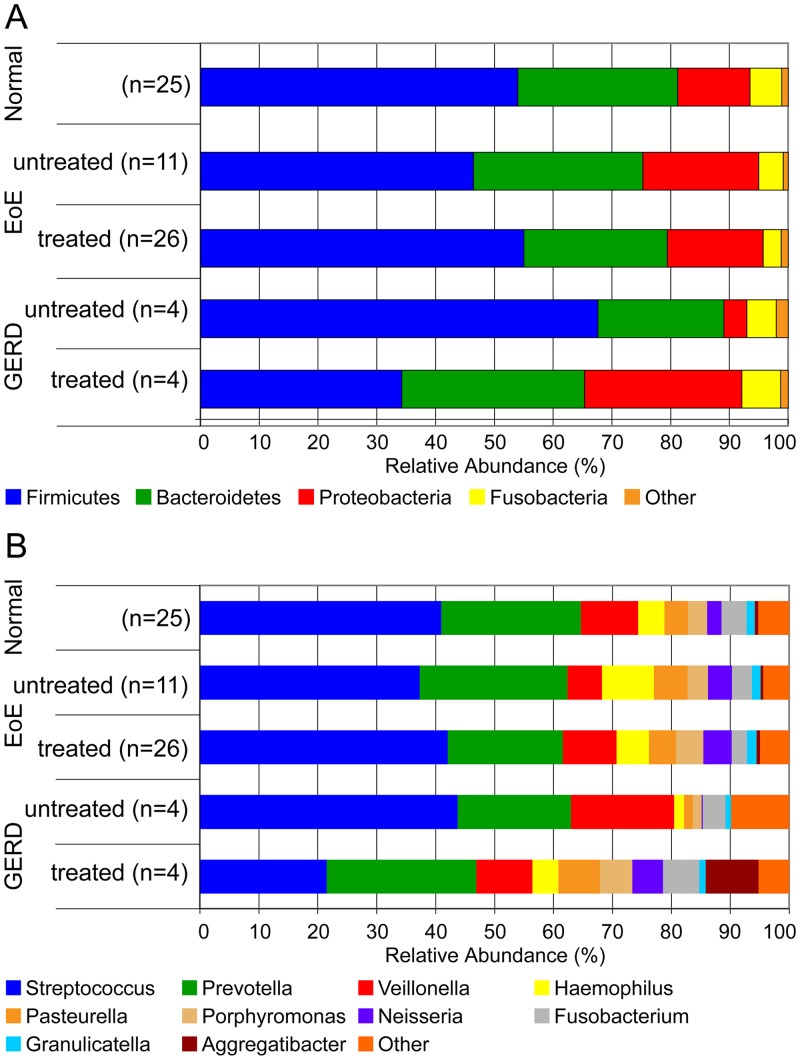
Esophageal phyla are similar in EoE compared to normal esophagus, and treatment affects the genus abundance in EoE and GERD. Abundance of esophageal phyla and genera as captured using the EST. A. Bar graphs present the aggregate of the relative phylum abundance on the ESTs of Normal subjects (n = 25), EoE subjects untreated (n = 11), treated (n = 26), and GERD subjects untreated (n = 4), PPI treated (n = 4). Each phylum is indicated in a different color. The width of the bar corresponds to the relative phylum abundance. B. Bar graphs present the aggregate of the relative genus abundance on the ESTs of Normal subjects (n = 25), EoE subjects untreated (n = 11), treated (n = 26), and GERD subjects untreated (n = 4), treated (n = 4). Each genus is indicated in a different color. The width of the bar corresponds to the relative genus abundance. The Other category includes taxa with <1% relative abundance in all samples.

We next determined the impact of inflammation and treatment on the bacterial composition in EoE. To accomplish this, we compared the bacterial taxa identified based on subject groups (Table 1) at the level of phylum and genus. Four phyla (*Bacteroidetes*, *Firmicutes*, *Fusobacteria* and *Proteobacteria*) were predominant, and similar, in active EoE, treated EoE and normal mucosa (Fig 2A).

In contrast to EoE, untreated GERD showed an increase in *Firmicutes* and decrease in Proteobacteria compared to normal subjects. Additionally, treatment of GERD with PPI was associated with an expansion of Proteobacteria. Comparisons of the predominant genera present in the different subject groups showed similar distribution with the exception of PPI treated GERD (Fig 2B). Treatment of GERD produced a community with similar relative abundance among the predominant taxa observed, without major changes to the genera present. The one exception is *Aggregatibacter* that was not detected in untreated GERD but expanded to a relative abundance of 18% in the PPI treated GERD group with a concomitant decrease of *Streptococcus* relative abundance from 47% in untreated GERD to 21% in PPI treated GERD. However, the relative abundance of *Streptococcus* was not decreased and *Aggregatibacter* was not increased in the other groups (normal and EoE) where half of the subjects were treated with PPI (Table 1) (Fig 2B).

**Fig 3:**
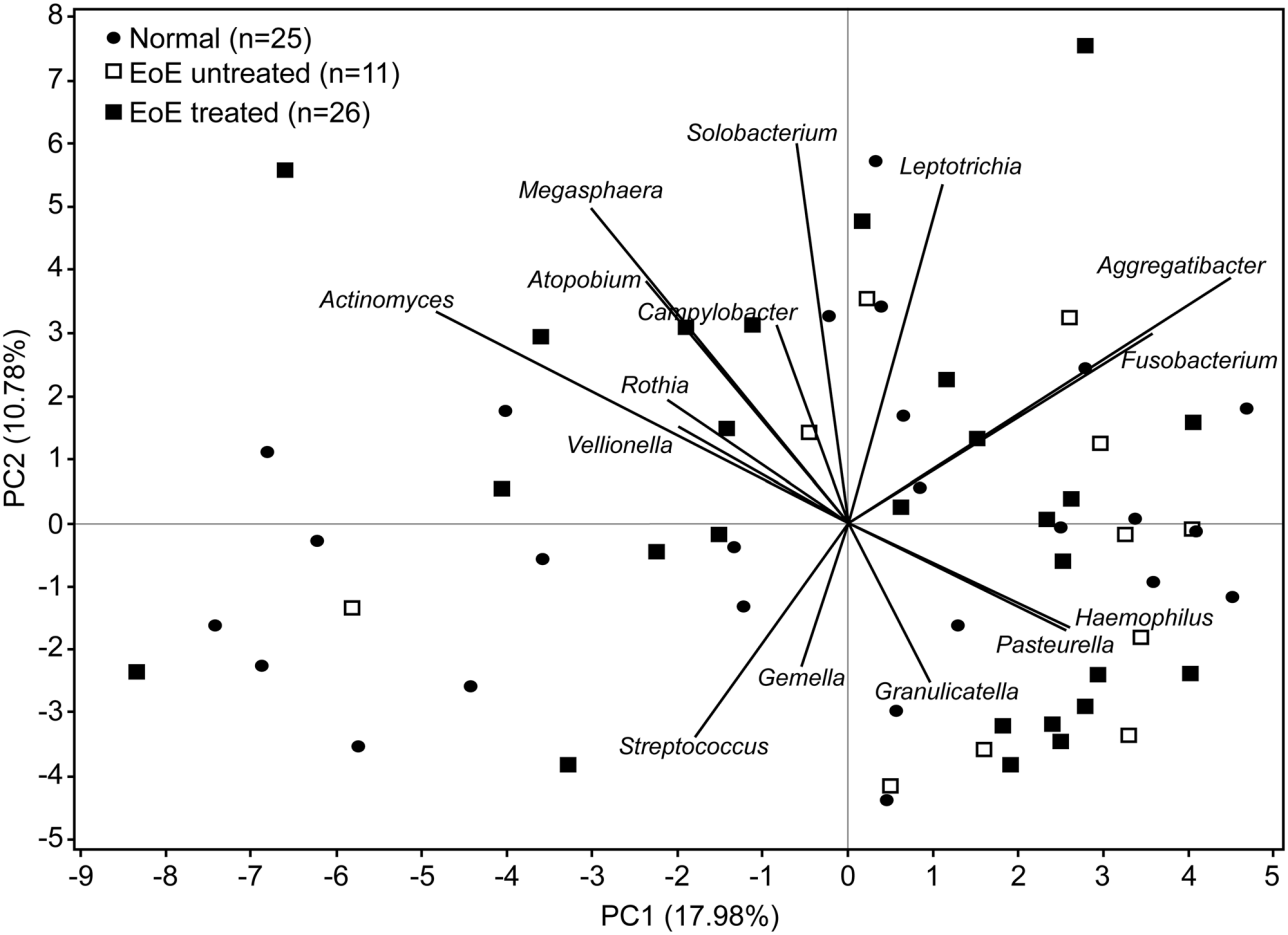
Principal Components Analysis (PCA) of Normal and EoE samples. PCA using normal esophagus (n = 25) and EoE samples (n = 11 untreated and n = 26 treated). The biplot displays the first two principal components which explains 29% of the variability across genera, as well as vectors corresponding to the weight and direction of the loadings for the highest weighted genera. Vectors pointing in the same direction are genera that are positively correlated; those going in opposite directions are negatively correlated.

Principal Components Analysis (PCA) focused on EoE subjects alone to examine the relationship between subjects based on communities present. The first two components are shown in Fig 3 along with vectors displaying the weight and relationship with the genera that have the highest contribution to these components. The first two components explain approximately 29% of the variability in the dataset. The EoE subjects are generally positive in PC1 corresponding to larger amounts of *Haemophilus*, *Pasteurella*, *Fusobacterium* and *Aggregatibacter* and smaller amounts of *Actinomyces*, *Veillonella* and *Rothia*. *Streptococcus* was negatively correlated with *Leptotrichia*.

**Fig 4:**
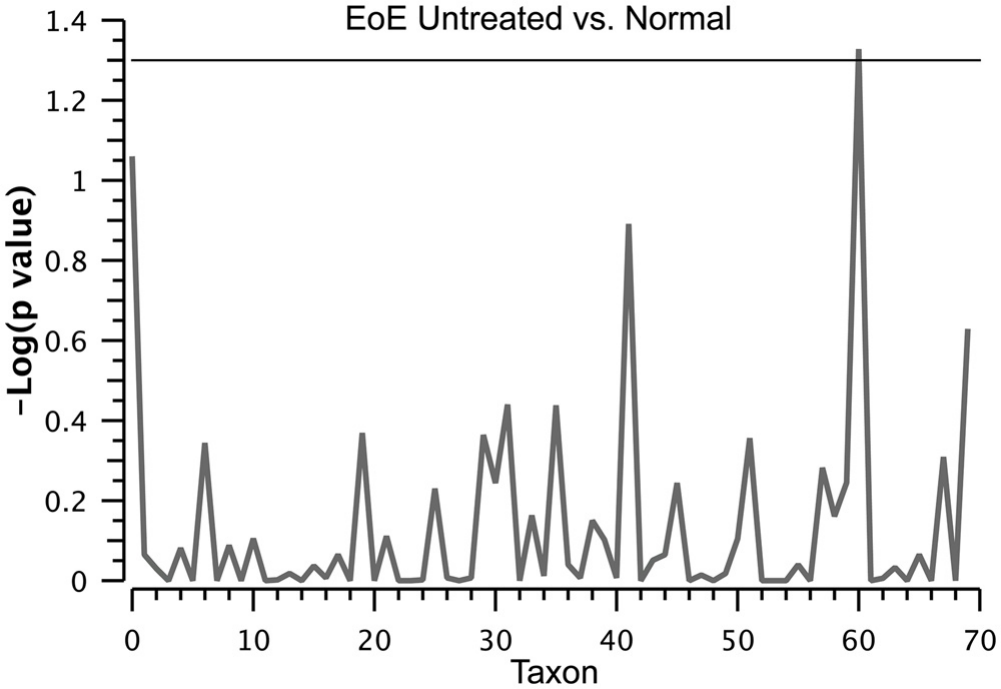
Manhattan plot of the two part statistical analysis of normal (n = 25) versus untreated EoE (n = 11). The plot shows the negative log_10_ p-value for each taxon identified. The single significant genus identified was *Haemophilus*, which was elevated in EoE subjects.

**Fig 5:**
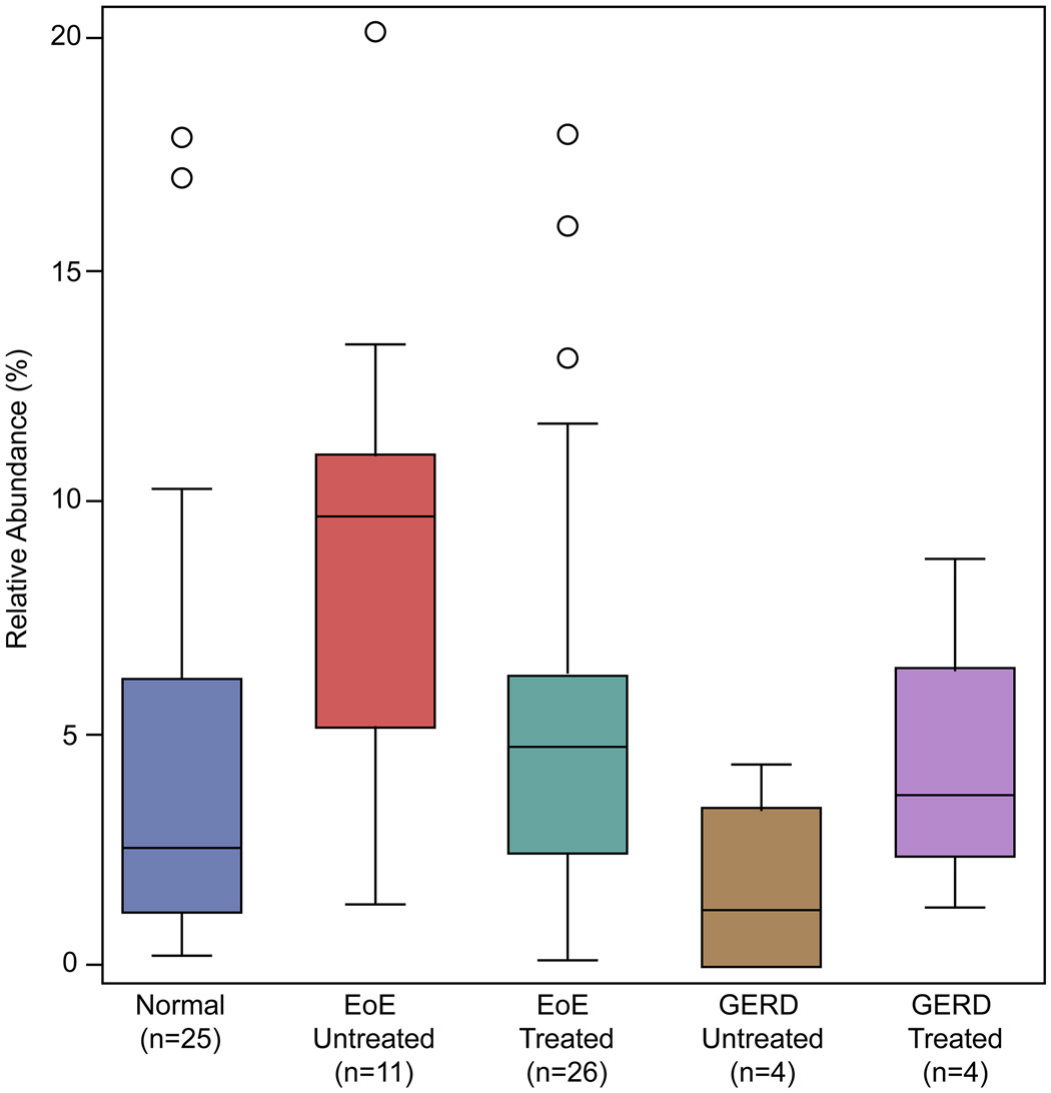
Comparison of *Haemophilus* across all subject groups. The relative abundance of *Haemophilus* for each group is plotted. Box represents median and 25^th^ and 75^th^ percentile (interquartile range, IQR) and whiskers represent 1.5x IQR. Individual samples outside the 1.5x IQR are marked as open circles.

We compared the untreated EoE and normal subjects using a two-part statistic to determine if any genera differed across these two groups. *Haemophilus* was identified as significantly increased in untreated EoE compared to normal control subjects (p = 0.047, Fig 4 and S1 Table). All subject groups were positive for *Haemophilus*, but the relative abundance was significantly higher in untreated EoE subjects compared to control subjects with a normal esophagus or GERD (Fig 5).

**Fig 6:**
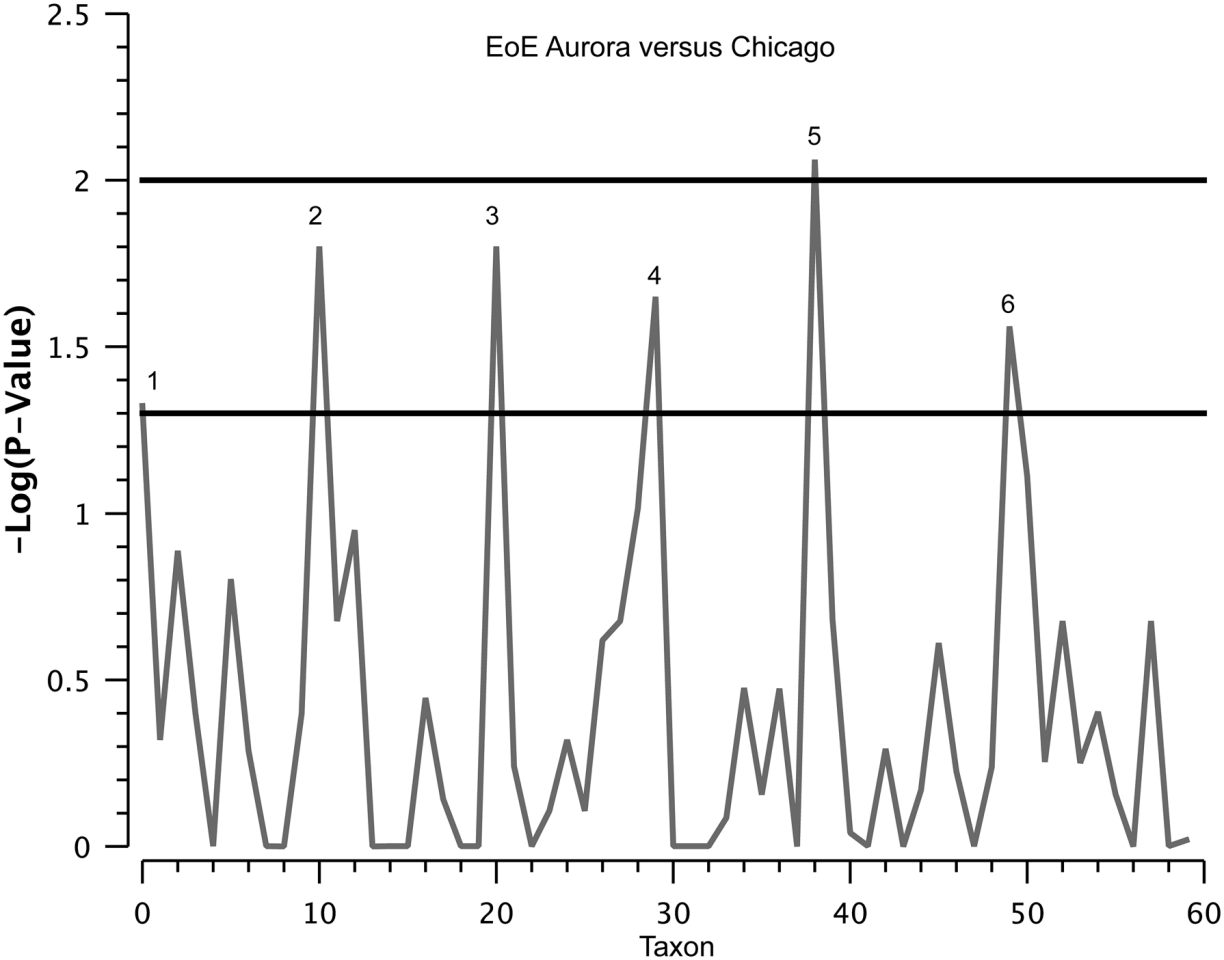
Manhattan plot of the two part statistical analysis of Aurora, CO (n = 17) versus Chicago, IL (n = 20) microbiome samples. The plot shows the negative log_10_ p-value for each taxon identified. There are six significant taxa identified. The peaks correspond to 1. *Actinomyces* (p = 0.048), 2. *Prevotella* (p = 0.016), 3. *Streptococcus* (p = 0.016), 4. *Parvimonas* (p = 0.022), 5. *Fusobacterium* (p = 0.009) and 6. *Aggregatibacter* (p = 0.027). All significantly different taxa were elevated in the Aurora relative to Chicago group except for *Streptococcus*.

We also observed a pronounced difference in EoE subjects between centers (Chicago and Aurora). While the microbiota in control subjects between centers were not significantly different (not shown), the comparison of EoE subjects in Chicago and Aurora demonstrated that, except for Streptococcus, all significantly different taxa were significantly increased in the Aurora relative to Chicago group (Fig 6 and S2 Table).

## Discussion

Here we report that the inflamed esophagus in EoE contains a significant increase in *Haemophilus*, and that this microbial pattern returns to that found in GERD and subjects with a normal esophagus following standard of care EoE treatment. In addition, we determined that epithelial eosinophilia did not seem to influence the load of bacteria, and that the microbiome measured in EoE subjects differed in Chicago and Aurora. Taken together, these results suggest that different mechanisms may influence the microbiome in EoE compared to that seen in other conditions, and that the geographical location of a subject and / or treatment may influence the microbiome in EoE.

The load of bacteria is increased in subjects with EoE and GERD, independent of the diagnosis, treatment, or disease activity in subjects with EoE, however the treated subjects may not be truly uninflamed at the time of sampling (Fig 1). However, the number of taxa and community composition was similar across all groups, indicating that increased load is associated with inflammation. The predominant taxa were similar in EoE subjects irrespective of the type of treatment, elimination diet or topical corticosteroid. However, subjects with GERD on PPI had a more consistent distribution of predominant taxa, and emergence of a specific proteobacteria lineage, *Aggregatibacter*, with a concomitant decrease in *Streptococcus* (Firmicutes). These findings are in agreement with a prior study that identified a decrease in *Streptococcus* in GERD [24], but the increase in *Aggregatibacter* in subjects on PPI has not been reported. The effect of PPI treatment on microbial communities is known for gastric fluids and esophageal tissues [25]. It has been suggested that PPIs may target certain bacterial proton pumps (P-type ATPases) typical of *Streptococcus* spp. PPIs may also affect the esophageal microbiota by increasing the pH of refluxed gastric contents [26]. However, the impact of PPI on *Streptococcus* and *Aggregatibacter* seems to be limited to the GERD group as the relative abundance of these genera were not changed in the normal or EoE groups.

The primary genus differentially identified in the untreated EoE subjects was *Haemophilus*, which contains known human pathogens such as *Haemophilus influenzae*. It is possible that the EoE group contains different community types that were not apparent in this analysis. The PCA analysis suggests that the majority of the variability across microbial compositions is not directly due to disease state or treatment, as most of the treated EoE subjects were similar in the first two principal components (Figs 2 and 3). However, there is a significant overlap of a subset of the subjects undergoing treatment with the untreated group; it is unclear if this represents differential response to treatment.

The disease activity in the esophagus does not seem to directly affect the load of bacteria in EoE. However, eosinophils have been described to possess numerous anti-microbial properties with the release of their granule proteins, defensins, and extracellular DNA traps [2–4]. The microbiota in untreated EoE subjects showed a shift from a mostly Gram-positive population (Firmicutes) to an increase in Gram-negative bacteria (primarily Proteobacteria) similar to what has been described in GERD [24]. Previous implication of Gram-negative bacterial involvement in reflux esophagitis [27] is consistent with the observed increase in Proteobacteria and *Haemophilus* in EoE.

These data suggest that treatment may impact the microbiota. Subjects at Children's Hospital Colorado, in Aurora, CO were treated a combination of topical corticosteroids and/or elimination diets, whereas subjects in Chicago, IL were treated mostly with food elimination diets. Diet is known to have an important effect on the lower gastrointestinal microbiota. Studies of diet-induced changes in the microbiota in the lower gut have shown that diet rapidly alters the gut microbiome [28–30]. However, the effect of diet on the upper gastrointestinal tract is largely unknown. We examined treatment in the current dataset, but additional subjects are needed to allow subgroup analysis capable of isolating the multiple approaches to clinical management of these patients. While corticosteroids and food elimination diets are standards of treatment in EoE, the current study was not sufficiently powered to inform the effects of individual treatments on the esophageal microbiota. In addition to clinical management, many factors impact the microbiome in children including the mode of child birth, early feeding methods, and environmental exposure [31, 32]. Future studies will require more detailed dietary information for analysis to better understand its impact on the variability in esophageal bacterial communities observed in EoE.

As we previously published, the esophageal microbiome detected by the EST is representative of the mucosal microbiome present on paired mucosal biopsies, and specific to the esophagus, which is significantly different from the oral and nasal microbiome [5]. However, the capacity for *Haemophilus* to enter the epithelial layer suggests that these organisms may be able to exploit the damaged barrier in EoE to help propagate and perpetuate inflammation in this disease [33, 34]. In the current study, we did not observe any significant differences in the esophageal microbiota based on age, gender or ethnicity, but additional studies with a larger number of subjects will be needed to more fully address these variables. Moreover, the gut microbiome has been shown to be stable by age 3 [35]. However, based on the cross-sectional design of our study, it remains unclear whether the changes we have identified in the esophageal microbiome contribute to the pathogenesis of EoE or are a consequence of EoE associated esophageal inflammation and remodeling. Longitudinal studies will be needed to address this question.

The minimally invasive EST collection approach provides the opportunity to obtain longitudinal samples, which will allow future monitoring of the microbiome during treatment of EoE. This will provide access for studying changes that occur in conjunction with specific interventions that induce disease remission or exacerbation. The capacity to obtain samples associated with particular treatments within individual patients will greatly assist our understanding of their effects by removing the inter-subject variability inherent in the microbiome.

This study is the first report to examine the microbiome in esophageal samples from patients with EoE. An increase in bacterial load was found, but there were limited differences between the bacterial community composition in treated and untreated EoE. However, patients with active EoE show a significant increase in *Haemophilus* in their esophagus. GERD subjects were included as a disease control, and while the numbers in this group were limited, there was a strong signal associated with PPI treatment. These data represent a baseline for bacterial communities in EoE subjects, and provide important findings for design of future studies, e.g. longitudinal analyses that follow changes in the microbiome during treatment-induced disease remission. Further studies in multiple centers with a larger number of subjects will help delineate the effect of alterations in the microbiome and treatment on esophageal inflammation, and potentially help to identify novel therapeutic targets for these diseases.

## Supporting Information

S1 Table Summary of two-part analysis for comparison between untreated EoE and normal subjects. The number of subjects positive for each taxon, proportion of positive subjects, and median relative abundance for positive subjects is reported.
(XLSX)

S2 Table Summary of two-part analysis for comparison between EoE subjects in Aurora and Chicago. The number of subjects positive for each taxon, proportion of positive subjects, and median relative abundance for positive subjects is reported.
(XLSX)
